# Evaluating Integrated Surveillance for Antimicrobial Use and Resistance in England: A Qualitative Study

**DOI:** 10.3389/fvets.2021.743857

**Published:** 2021-11-02

**Authors:** Houda Bennani, Laura Cornelsen, Katharina D. C. Stärk, Barbara Häsler

**Affiliations:** ^1^Veterinary Epidemiology, Economics and Public Health Group, Department of Pathobiology and Population Sciences, Royal Veterinary College, London, United Kingdom; ^2^Department of Public Health, Environments and Society, London School of Hygiene and Tropical Medicine, London, United Kingdom; ^3^Department of Animal Health, Federal Food Safety and Veterinary Office, Bern, Switzerland

**Keywords:** antimicrobial resistance, antimicrobial use, integrated surveillance, evaluation, One Health

## Abstract

Integrated surveillance systems for antimicrobial use (AMU) and antimicrobial resistance (AMR) require regular evaluation to ensure the effectiveness and efficiency of the system. An important step in the evaluation is to choose an appropriate tool for the purpose of the evaluation. The “Integrated Surveillance System Evaluation” (ISSE) framework is a conceptual framework that was developed to evaluate One Health (OH) integration in surveillance system for AMU/AMR. This study aimed to evaluate the performance and value of integrated surveillance system for AMU/AMR in England by applying the ISSE framework, which was used to develop data collection protocols and define the study design. A qualitative study using semi-structured interviews was conducted to collect the data and analyse it thematically. Eighteen stakeholders from human, animal, food and environment sectors that are involved in AMU/AMR surveillance were interviewed. Four main themes emerged from the analysis: (1) Cross-sectoral integration in the surveillance system for AMU/AMR; (2) Production of OH outputs and outcomes; (3) Drivers and barriers to cross-sectoral collaboration; and 4) Need for more cross-sectoral collaboration. The findings showed that there were links between integrated surveillance information, decision making and interventions. However, there were only few OH examples, such as the UK AMR contingency plan, where the potential of cross-sectoral collaboration was fully exploited. A lot of the benefits described were related to the generation of information and increase in knowledge and understanding without links to how the information generated was used. While these intangible benefits have a value on their own, being able to link surveillance information and mitigation measures would help to enhance the value of integrated surveillance. In terms of improvement, the main areas identified were the development of more harmonised methods for data collection and analysis, provision of resources dedicated to cross-sectoral collaboration, improved coordination, and collection of surveillance data from the environment and from companion animals. By identifying links between OH surveillance information produced and various outputs and outcomes; this study helped to understand the wider benefits of integrated surveillance for AMU/AMR in England and provided insights on how the system could be improved and efficiency increased.

## Introduction

The worldwide recognition of the crisis caused by antimicrobial resistance (AMR) has prompted international organisations to call for all countries to develop national action plans (NAP) to address this threat (1). Surveillance is an essential component of any NAP and due to the multiple transmission pathways of AMR, surveillance data need to be collected from humans, animals, food and the environment in a coordinated and harmonised way to allow cross-sectoral analyses (2). This integrated approach to surveillance is also known as One Health (OH) surveillance and has been promoted by international organisations (1). One Health surveillance for AMU and AMR refers to surveillance that is based on a systemic, cross-sectoral, multi-stakeholder perspective to inform mitigation decisions with the aim to keep antimicrobials effective for future generations.^1^ Integrated surveillance across the different sectors for antimicrobial use (AMU) and AMR allows a better understanding of the sources of infections and the routes of transmission; therefore enhancing our understanding of the epidemiology of AMR and informing better evidence-based interventions. In addition, it allows to link data on AMU and AMR, which would enable to identify if the reduction of AMU is having the desired effects on AMR in both humans and animal populations (2).

To ensure that the surveillance systems are operational, efficient and cost-effective, regular evaluation is needed (3). Even if the system is not built to follow OH surveillance principles, evaluation from a OH perspective allows to assess whether cross-sectoral collaboration generates added value (4). An important step in the evaluation is to choose an appropriate tool for evaluation to ensure that the outputs are reliable for stakeholders and decision makers (5). To the knowledge of the authors, only one framework exists that was developed specifically to assess the integration of the surveillance system for AMU/AMR, called the “Integrated Surveillance System Evaluation” (ISSE) framework (4, 6). This framework was developed based on the Canadian Integrated Program for AMR Surveillance (CIPARS) and provides a conceptual basis for structuring the evaluation of different surveillance outcomes.

Multiple tools and frameworks are available either for the evaluation of surveillance (e.g., SurvTools developed in the Risk-based Animal health Surveillance Systems-RISKSUR- project, the Assessment Tool for Laboratories and AMR Surveillance Systems-ATLASS) or One Health (e.g., the Network for the Evaluation of One Health -NEOH tool). These tools range in application from assessing laboratory capacity in the surveillance system to the assessment of governance, but none of them provides a focussed approach for the evaluation of OH AMU/AMR surveillance. A recent study documented the strengths and weaknesses of six evaluation tools namely, SurvTools, NEOH, ISSE, ATLASS developed by the Food and Agriculture Organization (FAO), the Progressive Management Pathway tool on AMR (PMP-AMR) developed by the FAO, and the Evaluation of Collaboration for Surveillance (ECoSur) tool (7). These tools were applied to a range of case studies conducted in eight countries. The authors found that the tools covered aspects of AMU/AMR surveillance and OH to varying degrees and they presented high degree of complementarity. PMP-AMR, ATLASS, ECoSur and NEOH are evaluation tools that provide a scoring system to obtain semi-quantitative results, whereas ISSE and SurvTools would result in a plan for how to conduct evaluation(s). The NEOH and ISSE were perceived as the best tools for evaluation of OH aspects, and ECoSur as best for evaluation of the quality of collaboration (7). Depending on the evaluation questions, assessors will need to select a tool that suits their needs (7). To guide users in choosing a suitable evaluation tool, an international network of scientists developed guidance for choosing an assessment approach from an inventory of tools suitable for evaluating integrated AMU and AMR surveillance systems in the project “Co-Eval-AMR–Convergence in evaluation frameworks for integrated surveillance of AMU and AMR.”^1^

In the UK, a five-year AMR strategy based on a One Health approach was developed in 2013 and included actions relevant to humans, animals and the wider environment. In 2019, a nationwide OH NAP was developed alongside a 20 year vision for containing and controlling AMR by 2040 (8, 9). Surveillance is an integral part of the NAP and the data collected include AMU data from humans and animals and AMR data from humans, animals and food (10). The surveillance system for AMU/AMR in the UK is organised in each sector separately with presence of cross-sectoral integrated activities (10). The aim of this study was to evaluate qualitatively the performance and value of the integrated surveillance system for AMU/AMR in England by applying the ISSE framework. This framework was selected, because it is the only one developed specifically to evaluate integration in the surveillance system for AMU/AMR. The focus is on England and not the whole UK because our previous mapping of the surveillance system for AMU/AMR in the UK showed variations in the surveillance system between the four UK nations (10). To our knowledge this is the first evaluation of the AMU/AMR surveillance in England from a OH perspective. Information about the performance of the integrated surveillance system for AMU/AMR can provide insights into what is working well in the system, where are gaps and how the system can be improved and efficiency increased.

## Methods

### Conceptualisation of the Evaluation

**Figure 1:**
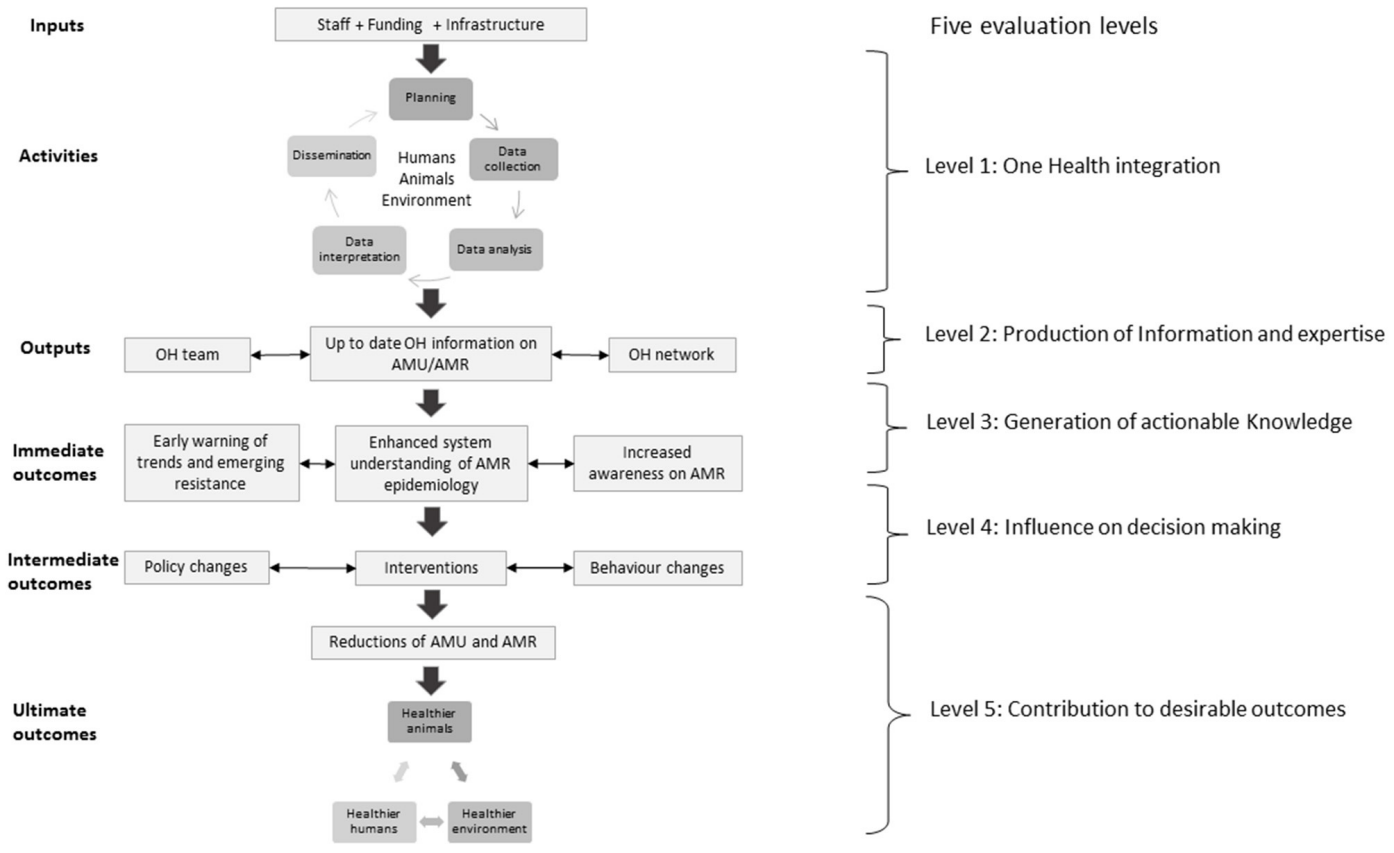
Logic model for a generic OH surveillance system for AMU/AMR including five evaluation levels. Adapted from Aenishaenslin et al. (4).

The ISSE logic model for OH surveillance for AMU/AMR was used as a basis to conceptualise the evaluation of the integrated surveillance system for AMU/AMR in England and to develop data collection protocols. It comprises of five evaluation levels based on the hierarchy in the expected chain of events that emerged from focus group discussions and workshops organised with CIPARS team members (4, 6). The logic model (Figure 1) illustrates the relationships between the different integrated surveillance activities, outputs and the expected outcomes and impact. A modification was made to the logic model by adding a planning step to the surveillance activities. The different levels of the evaluation target different aspects in the system that include: (1) OH integration across the different components of the surveillance system including data collection, analysis and interpretation, and information dissemination; (2) Production of OH information and expertise; (3) Generation of actionable knowledge; (4) Influence on decision making; and (5) Contribution to desirable outcomes.

Possible benefits of integrated surveillance depend on the links between the information generated by integrated activities and changes implemented to reduce AMR (e.g., an intervention or new policy). In the ISSE framework, the value of integrated surveillance for AMU/AMR was conceptualised across immediate, intermediate and ultimate outcomes (4, 6). Immediate outcomes include increased understanding of AMR epidemiology from a OH perspective and early warning of emerging resistance. Intermediate outcomes include changes in policy or behaviours resulting from OH information generated, and the expected value is in the size of the reduction in AMU and AMR that results from these changes. Ultimate outcomes include tangible benefits such as improved animal, human and environmental health and associated socioeconomic benefits (4, 6).

We also made use of two other evaluation tools to expand on OH integration, namely the NEOH (11) and the ECoSur (12) tools. The NEOH tool provides guidance for the evaluation of OH initiatives including description of the context, the initiative, its theory of change, identification of outcomes and assessment of One Health-ness (i.e., the strength of OH). The latter is assessed using six characteristics that include systemic thinking, holistic planning, transdisciplinary working, OH sharing, OH learning, and systemic organisation (11). The ECoSur tool provides guidance on how to assess the organisation, functioning and functionalities of collaboration taking place in a multi-sectoral surveillance system (12).

### Study Design

Qualitative interviews were conducted using a semi-structured interview guide (Supplementary Material A) with open-ended questions that enabled depth and flexibility in exploring experiences, influences and opinions of respondents.

The guide was developed to capture data on (i) integrated surveillance activities for AMU/AMR in England (the activity was considered integrated if there was collaboration between at least two of the following sectors: animal, human, food and the environment), (ii) links between surveillance activities and outputs, (iii) links between outputs and outcomes, (iv) the impact of surveillance information on decision making, (v) the impacts of the decisions attributable to integrated activities, (vi) data and information sharing, (vii) need for improvement. Questions (i) to (v) were developed by the authors following the logic of the ISSE framework to explore the links between the integrated surveillance activities and the outputs and outcomes. To account for data on OH sharing [questions (vi)], questions related to the evaluation of data and information sharing were included based on protocols from the NEOH framework (11). Because effective coordination underpins all surveillance activities, questions related to the steering and coordination of integrated surveillance activities were added to questions (i) based on the ECoSur tool (12).

### Data Collection

In a previous study to characterise and map the surveillance system for AMU/AMR in the UK, a list of key organisations involved in the national surveillance system for AMU/AMR was developed (10). Interviewees were purposefully selected from this list and also using snowball sampling (i.e., participant referrals of other participants). The key informants were selected because they had knowledge and expertise on AMU/AMR surveillance in England and were directly involved in the surveillance system. Selecting participants who were able to provide rich and in depth information about the questions researched was critical for the study. Participants included representatives from regulatory bodies, implementing bodies and other stakeholders from human, animal, food and environment sectors. The list of these organisations is presented in Table 1 with the number of participants from each organisation included in brackets. Information about the roles of participants is included in Supplementary Material B. Two pilot interviews were conducted to test the interview guide. Unclear questions were modified accordingly based on the feedback provided. The interviews were conducted by HB between May and December 2019 (eleven in person, three by phone and four by skype) and lasted for up to 60 min. An information sheet with the description of the project and the objectives of the interview were sent to participants prior to the meeting and they had the possibility to clarify any questions before the interview. Written consent for participation in the interview and audio recording was obtained from participants before the interview. Interviews were recorded with a digital device (Olympus WS-853 MP3 digital stereo voice recorder). The findings were anonymised to protect the identity of informants and participants were coded as “Pn,” where n ranged from 1 to 18. The interviews broadly followed the topic guide and although the topics were common across interviews, the order and emphasis on different themes varied to focus on the issues most relevant for each participant.

**Table 1:** Grid for participants' selection.

	**AMU and AMR surveillance in England**			
	**Humans**	**Animals**	**Food**	**Environment**
**Policy and regulatory bodies**	Public Health England (PHE) [3]	Veterinary Medicines Directorate (VMD) [1]	Food Standards Agency (FSA) [3]	Environment Agency (EA) [1]
**Implementing bodies**		Animal and Plant Health Agency (APHA) [3]		
**Other sectors/stakeholders**	- Animal industry: Responsible Use of Medicines in Agriculture Alliance (RUMA) [1] - Small animal sub-sector^*^: VetCompass [1], Small Animal Veterinary Surveillance Network (SAVSNET) [1] - Academia/Research: Bristol University [1], Quadrum Institute [1] - Veterinary body: British Veterinary Association (BVA) [1] - Others: Cornwall One Health AMR group [1]			

*The number of participants from each organisation is included between brackets*.

* *These participants were from Academia and were interviewed because of their expertise in VetCompass and SAVSNET*.

This study received ethical approval from the Social Sciences Research Ethical Review Board (SSRERB) at the Royal Veterinary College, with the approval number URN SR2019-0204.

### Analysis

All interviews were recorded and transcribed verbatim by a professional company. Transcripts were transferred into NVivo12 Plus (QSR International Pty Ltd) for data management and analysis. A thematic analysis was used in this study, which is a method for identifying, analysing and reporting patterns (themes) within the data (13). A combined approach to the analysis was used enabling themes and subthemes to be developed both deductively from the logic model and the interview topic guides and inductively from the experiences and views of participants. Initially, four transcripts were analysed by the first author and codes were generated. Then, a meeting with a second researcher in the team (BH) was organised to review the emerging codes and discuss the interpretation of the findings. Following this discussion, the coding frame was updated and then applied iteratively to the transcripts, refined and reapplied until no new codes were generated. Key themes identified are used as headings to organise the findings and summarised thematic information from all participants were included and the most illustrative quotes from the interviews were used to highlight critical points.

## Results

**Figure 2:**
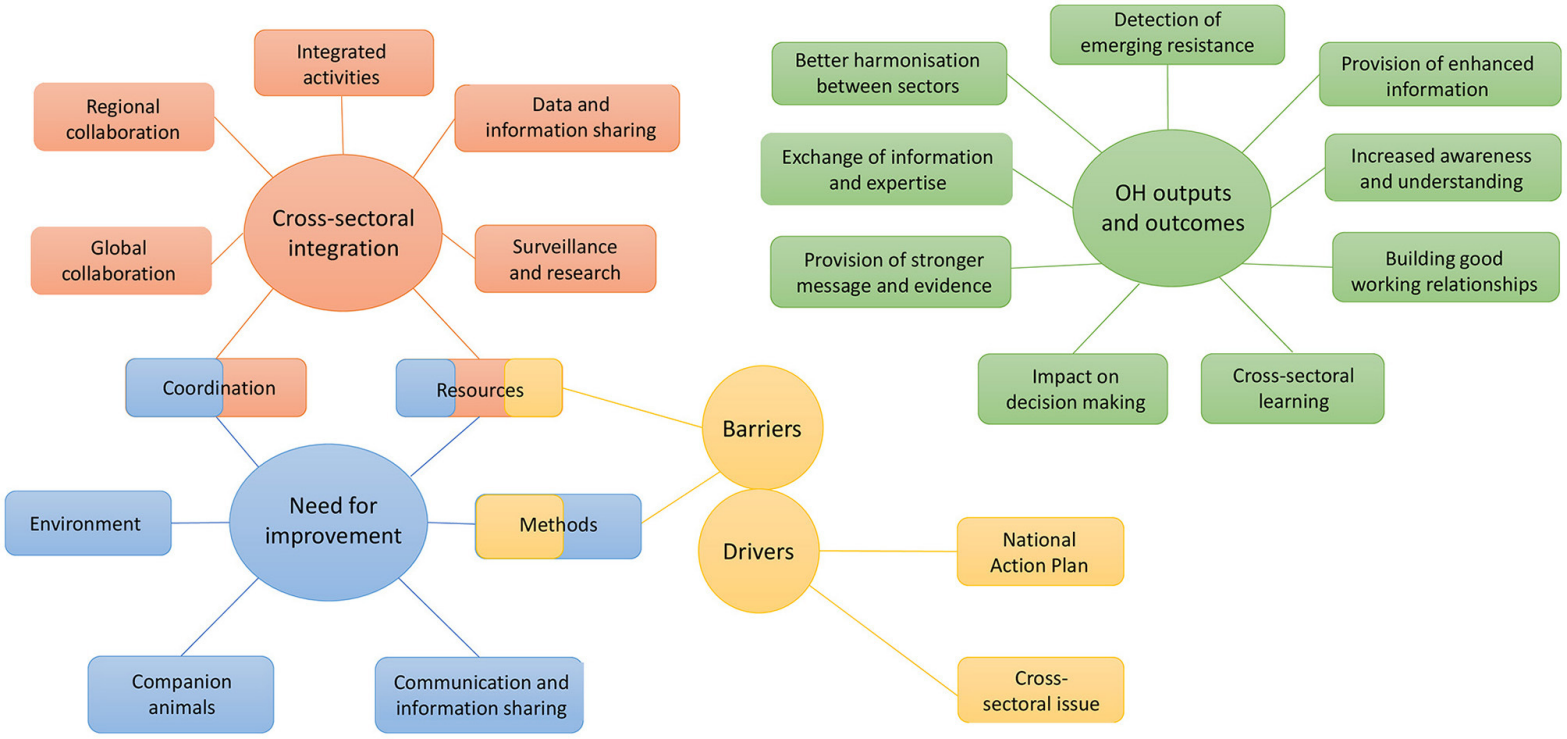
Overview of themes (circles) and subthemes (rectangles) developed during the analysis of the interviews with 18 stakeholders.

In the following sections, the key themes identified are presented, namely (1) cross-sectoral integration in the surveillance system for AMU/AMR, (2) production of OH outputs and outcomes, (3) drivers and barriers to cross-sectoral collaboration, and (4) the need for more cross-sectoral collaboration. An overview of the themes and subthemes is presented in Figure 2.

### Cross-Sectoral Integration in the Surveillance System for Antimicrobial Use and Antimicrobial Resistance

Integrated surveillance activities reported by interviewees are described below, in addition to the following sub-themes identified: data and information sharing, coordination of cross-sectoral collaboration; resources; global and regional collaboration; and the link between surveillance and research.

#### Integrated Surveillance Activities

Various cross-sectoral surveillance activities were reported by the interviewees and several of them are UK wide, even though the evaluation focused on England. Key integrated activities are presented in Table 2 and more detailed descriptions of these activities can be found in (Supplementary Material C). Examples of other integrated surveillance activities reported by participants include collaboration between the two main government (PHE and APHA) laboratories to exchange information about cases and compare isolates, joint conferences, and collaboration between APHA and the British Society for Antimicrobial Chemotherapy (BSAC).

**Table 2:** Integrated surveillance activities.

**Integrated activity**	**Description**
AMR contingency plan or Res-Alert	Refers to the response upon identification of a resistant bacterial isolate from an animal considered to pose a potential risk to human and/or animal health. It is a UK wide initiative initiated in 2015 and coordinated by the VMD in collaboration with government agencies covering human, animal, food and the environment, and the Devolved Administrations.
UK OH report on antibiotic use and resistance	Produced by PHE and the VMD. Published for the first time in 2015 with and a second report published in 2019 (14, 15).
Advisory committees in one sector with representatives from other sectors	Examples of these committees include the DEFRA antimicrobial resistance coordination (DARC) group; the Advisory Committee on Antimicrobial Prescribing, Resistance and Healthcare Associated Infection (APRHAI); and the Advisory Committee on Microbiological Safety of Food (ACMSF) subgroup on AMR;
The Responsible Use of Medicines in Agriculture Alliance (RUMA) Target Task Force (TTF)	This group was formed in 2016 to develop specific targets for the key UK livestock sectors. The group comprises mainly stakeholders from the animal health sector representing the government and industry, with only one representative from the food sector which is the FSA. In addition, an independent scientific group was formed with experts from animal and human sectors to provide advice to the TTF group.

#### Data and Information Sharing

In England, there is no annual joint surveillance report for AMU/AMR but there are annual surveillance reports published separately by human, animal and food sectors that are publicly available online (16–18). For animals, these data are published aggregated for the UK in the Veterinary Antimicrobial Resistance Surveillance (VARSS) reports. In humans, there is the English Surveillance Programme for Antimicrobial Use and Resistance (ESPAUR) report for England. Also, indicators for AMU and AMR are publicly accessible in the “Fingertips” data portal^2^ which contains data on a range of public health issues categorised as “profiles;” one of them is “AMR Local Indicators.” For AMR in food (at retail level and from processing plants), results of the EU harmonised surveillance and reports of *ad-hoc* surveys conducted are published in the FSA webpage (10).

Although there is no structured mechanism for data and information sharing between sectors, respondents explained that a lot of the data generated are available in these public domains and accessible to all. Some of the data are also published in scientific publications and respondents highlighted that some sensitive data cannot be shared such as those related to the farms. The benefits of making the data widely available was also emphasised: “*Our overarching policy is to share as much data as possible, because the more people who have access to it the more other researchers can benefit and possibly find a solution for us” (P6)*.

In addition to the data that are publically available, one respondent mentioned that if some other data are needed, they could be requested and obtained: “*You mean requested or really have it? We could request it …There are lines of communication, but not on a routine basis”* (P10). There are also situations where certain agreements are made to share information depending on the project: “…*you have a lot of work going on in terms of research, for example, and as part of those research projects probably they have agreements to share data specifically for a particular pathogen or particular organism that they are looking at… Other than that, as part of Res-Alert I think it would be expected that sharing is kind of agreed”* (P1).

#### Coordination of Cross-Sectoral Activities for AMU/AMR Surveillance

The surveillance system for AMU/AMR is a key component of the NAP for AMR and the recommendations from the NAP are used as levers to implement collaborative surveillance activities. The NAP is coordinated by an interdepartmental High Level Steering Group (HLSG) which is dedicated to strategic decisions at high policy level but there is not a committee in charge of the coordination of integrated surveillance activities for AMU/AMR: “…* The national action plan is One Health. So the governance of that is going to be One Health with representatives from across the One Health spectrum. Then anything under that will still be One Health”* (P1). Although, there is a call in the NAP for more coordination and harmonisation of surveillance schemes across sectors, there is no clear guidance on how to organise collaboration across institutions to achieve this (9). Respondents explained that the different institutions need to define the modalities to achieve the objectives of the NAP including the organisation of cross-sectoral activities and that there are cross-sectoral groups and committees where discussions about these aspects take place: “*While we deliver the NAP and the vision of the NAP, everything is One Health, and from governance of the NAP, which at the moment we are in the process of developing as a result of the new action plan…., we have multi-sectorial groups and committees, for example, where we all work together as part of a cross-government One Health approach”* (P1). Examples of these groups include the different advisory committees in each sector such as the DARC group, the APRHAI and the ACMSF subgroup on AMR. Most participants mentioned the importance of cross-sectoral collaboration on AMU/AMR surveillance to tackle AMR and some mentioned the lack of coordination or the need for improvement in coordination. These aspects are explored in more depth in section Need for More Cross-Sectoral Collaboration on AMU/AMR Surveillance.

#### Resources for Cross-Sectoral Collaboration

In terms of resources, respondents mentioned that there is no fund available specifically for cross-sectoral collaboration and that the budget available for surveillance needs to be used to cover any integrated activities: “*There is a budget for AMR surveillance and that budget should cover any Res-Alert” (P1)*. In cases where there are projects covering issues related to different sectors, then there is a budget to look specifically at that particular issue like for example in the case of ESBL: “*So generally if there's a cross sectoral issue, so ESBL is probably quite a good one. So ESBL E-coli in animals and men, so the Department of Health funded a study that has looked at ESBL occurrence in humans and in sewage and in farms in five regions of the UK*” (P4). This sectoral allocation of resources is due to how the sectors evolved historically into segregated “silos” (19). This siloed funding has been discussed in previous studies as a barrier to cross-sectoral collaboration (20, 21). Where cross-sectoral collaboration requires establishment of new structures and processes (e.g., creation of a new One Health unit or integrated database), a financial cost is involved. In the absence of dedicated funding, cross-sectoral collaboration relies on the use of existing infrastructure and in-kind contributions, which limits its potential.

#### Global Collaboration and the Value of Collective Data

The UK contribution to European and international surveillance programmes has been praised by respondents who highlighted the importance of comparing data to other countries and assessing them at global level, which add value to the data. The following quote illustrate this: “…*so within Europe, EFSA is the regulatory body for the animal side and ECDC the humans, so they do write joint reports, so all member states will submit to them and they will write joint reports and that gives you an idea of what may or may not be going through and the things that might be, so they do a lot of work to look at it at that level, which is a higher level than specific country levels*” (P5). These collective data allow to see the bigger picture, which enables to generate more knowledge and information.

Due to the uncertainties around Brexit, participants expressed concerns about the impacts that this could have on UK contribution to European surveillance and stressed that it is important that surveillance data from the UK are represented in the European picture: “*Final thing is obviously about data going forward in Brexit….We would hope that data on AMR will continue to be collated by EFSA and others so I think there is a challenge there to make sure that the UK surveillance data goes into the international picture because we are part, we're still in Europe even if we're not in the EU. And that data collectively is important. ….We contribute our data to that but obviously we would feel, yes, this data needs to be kept intact because once you start to fragment it you're going to lose this information*” (P6). The EU implemented additional AMR surveillance requirements from 2021,^3^ but as these came into force post-transition period, the UK continues to follow the previous protocols. The extent of future data-sharing between the UK and the EU has not yet been agreed.

#### Regional Collaboration

One special type of collaboration that was identified was the Cornwall OH Antimicrobial Resistance group (CARG). CARG is the first regional OH group on AMR established in the UK and was set up in 2014 to ensure the implementation of a coordinated wide response to the AMR strategy in Cornwall (22). The group has representation from different stakeholders including doctors, veterinarians, microbiologists, nurses, dentists and researchers. Various activities conducted were mentioned including the organisation of OH AMR conferences in Cornwall with attendance from different stakeholders, organisation of educational sessions, and collaboration with veterinarians (mainly those working with dairy farmers as this is the biggest food producing sector in Cornwall) to collect and share antibiotic usage data. This collaboration was considered to have been successful the first year but then got very little interest from veterinarians the following years. When asked about the reason for this initiative being unsuccessful, P13 said: “*A real barrier to these groups being set up nationally is that there's no funding for them*.”

#### Surveillance and Research

An important integrated activity that was mentioned by most interviewees was the collaboration in research, with a respondent suggesting that there is more of a OH approach in research. Surveillance and research were seen to complement each other: “*Research underpins our surveillance because the methods we develop for research is used for surveillance”* (P5). An example that was mentioned was about some techniques that might be expensive, such as some molecular techniques, and cannot be implemented at a large scale. In this case, a target population can be identified through research and then surveillance data are collected from this population. Research provides also evidence that underpins AMR policy: “*We don't know yet in terms of the development of resistance and how much of this is caused by or starts in the environmental or animal sector, so there is still room for, or there needs to be much more research to understand this and where intervention would be most efficient or effective”* (P10). An emphasis was made to the difference between the research conducted by government laboratories or institutions with links to policy makers and those conducted by academia with the former having greater impact: “…*because we're working in government, that link to government is much more direct than it is in academia. Academia does do a lot of research….so it's helping to drive forward knowledge and understanding which will eventually impact,…but maybe some of the time it may not be as direct*” (P5).

### Production of One Health Outputs and Outcomes

This section outlines the various outputs and outcomes of OH surveillance activities that interviewees identified. These include the detection of emerging resistance, provision of better information for decision making, behaviour changes, exchange of information and expertise, learning from each other, increase of awarness and understanding, better harmonisation, provision of stronger messages and adding value to the data.

#### Detection of Emerging Resistance and Provision of Enhanced Information for Risk Assessment Leading to Better Risk Management and Decision Making

The AMR contingency plan (Res-Alert) allows to detect emerging resistance and was considered as an effective and action oriented activity: ”… *it works really well I would say and it is really … action and outcome orientated. They really get the right people …”* (P10). When a hazard is detected, relevant advisory committees are notified by the relevant agencies and meetings are organised to discuss the risk assessment and decide on the options for risk management and the plan for risk communication (14). During these meetings, decisions are taken by the OH team to generate the evidence needed to assess the risk of the particular hazard. One of the cases mentioned that triggered a Res-Alert was the discovery of mediated colistin resistance (mcr-1) in a pig farm in England in 2015 (23). In this case, various surveillance activities have been triggered in the different sectors including testing archived samples in humans and animals. The OH information collected was used to inform the multi-sectoral discussions and assessments conducted as part of the Res-Alert: “*I think the colistin…. So that was one of the major cases that we have had to deal with as part of Res-Alert…. Basically, after it was detected, we increased the surveillance, so we decided to do targeted surveillance and look at samples that were already stored in our archives to look back and see what was the prevalence, or whether it had been there before….So yeah, there were certain decisions that were made to actually generate the evidence that was needed to evaluate, to assess the risk that we were dealing with. One of them was to look at samples that were already available. To go to the farm where the colistin resistance was detected and do further sampling, collect data”* (P1). The outcomes of the assessments were shared with relevant stakeholders, which led to behaviour changes in farmers and voluntary restrictions on colistin use in food producing animals in the UK (14, 24). Public Health England declared that the risk to human health was considered to be very low and the FSA declared the risk to public health from food to be very low when food was properly handled and cooked (24). Because of timely risk assessment and data integration, better information was available which allowed better informed decisions to be taken.

#### Better Harmonisation Between Sectors

There are variations in the methods used in the various sectors and the work by the OH team producing the UK OH reports on antibiotic use and resistance (OH information) was seen as an opportunity for cross-sectoral discussions on ways to align the data across sectors. These reports included also recommendations to address data limitations and to improve integrated analyses. The following quote illustrates this: “…*we were having regular meetings with VMD and then there was DEFRA involved as well and so we were talking about it and thinking forward how things could work together, so we do work across sectors to try and get things to be aligned a bit better so that we can make the report more useful to everyone*” (P11). Another respondent explained how the work on these reports helps to improve the harmonisation of the methods used between sectors: ”…*since the publication of the first report, we worked on trying to harmonise more, so in the first report made recommendations like for a more representative sample of campylobacter in the human sector and we panel tested whether that could be more, be standardised across the two sectors*” (P10).

#### Exchange of Information and Expertise Leading to an Increase in Awareness and Understanding, Learning and Building Good Working Relationship

The DARC group was considered by respondents as a good platform for exchange of information and expertise between members of the OH team of DARC. DARC was also seen as a forum to discuss any issues raising from a OH perspective considering all the implications: “*One of the things a DARC would do is look at that and discuss this is obviously a threat, a hazard, to the UK, to the UK's poultry production. We know it affects humans. So if we get cases should we treat it? Should we try and contain? Should we try and eradicate it? So the decision doesn't just go to DARC because there are some salmonella committees as well, but then the decision can be discussed between us and medical colleagues*” (P4). Although DARC was not considered to be a decision-making group, it was felt that if an important issue was raised this would be followed up: “*I think DARC is mostly an advisory group so it's not a decision making group, although all the people who are involved in DARC have some sort of decisional power over this subject. So clearly if it's raised then it's agreed collectively that it would be good to do one thing or the other then normally it gets followed up*” (P2). Respondents acknowledged that the various sectors have different objectives and priorities but cross-sectoral communication was seen as key to enable reaching realistic solutions: “*I would say that each sector takes the lead in its own area. I think it's probably quite understandable…. So I would say a little bit of a delineation of responsibilities but often the discussion helps in trying to formulate what's a realistic policy” (P4)*.

Another outcome of cross-sectoral collaboration that was mentioned was the increase in understanding of how other sectors work and the different issues they are dealing with; learning from each other; and the establishment of good working relationship across sectors: “*It's understanding really because it's something that I'm certainly not an expert in and knowing that there are differences, getting a better understanding of how antibiotics are used in the environment or in agriculture in any way, which I think, I think that's important because it's something that you feel very isolated when you're looking at hospitals primarily, …, to know about the different influences outside and I think it's really useful to have this collaboration and knowledge…. And, it's a good working relationship that you build with other departments essentially*” (P11). Several examples about opportunities to learn from each other were mentioned by respondents such as benchmarking antibiotic use in humans and its impact on behaviour change in humans; it was suggested that this is something that could be applied to the veterinary sector. Benchmarking antibiotic use between pig farmers is possible due to the electronic Medicine Book (eMB) launched in 2016, which is a digital system to collect data on antibiotic use at farm level (25). A new centralised database called the Medicine Hub has also recently been launched for ruminants, which allows producers to record their antibiotic usage data and it has also a benchmarking facility (25).

#### Provision of Stronger Message and Evidence to Inform Interventions

OH information generated by integrated surveillance activities was seen to provide an understanding of the bigger OH picture and to generate stronger evidence to inform interventions: “*So I think it kind of gives you a much more rounded picture…. I suppose if you can show that you've considered sort of all areas, the veterinary and the human aspects and the environment, then it provides a sort of better evidence base for any recommendations that you put in there*” (P12). Similarly, activities that were conducted in a collaborative way were considered to provide more powerful messages: “*One of the key things with collaboration is that when you come out with your recommendation, if it says the human side, the veterinary side and the environmental side, everybody saying the same thing, that is an overwhelmingly powerful message, as opposed to the vets saying it or just a human saying it*” (P16). An example described by respondents were leaflets on Livestock Associated Methicillin resistant *Staphylococcus aureus* (LA-MRSA) produced collaboratively by different organisations including VMD, APHA, FSA and PHE.^4^,^5^ These leaflets were addressed to farmers and people working in abattoirs with information about the steps they could take to reduce the risk of infection: “*As you can see in the leaflet, lots of people collaborated in putting that together, so it's been signed by different government departments and different organisations*” (P1).

Sharing surveillance data to be used by other stakeholders was also seen as making them more powerful and adding value to the data: “*Well I think it makes it more powerful, I mean there's the factor of using this data in ways that may not be otherwise utilised. I think that provides benefits for the surveillance data and the partners within the project on both sides*” (P18).

RUMA's Independent Scientific Group, which was formed by experts from different sectors to provide sounder advice to the group, was also mentioned: “*We've built it because we need to have expertise in every area of this very complex subject and we then base all our actions and all our policies on sound science*” (P14). The RUMA'S TTF was considered as a successful initiative with most of the targets set forecasted to be met by 2020. These targets were developed by the TTF and focused not only on reduction in antibiotic use but also development of improved data collection systems for antibiotic usage at farm level, improved husbandry and biosecurity practises, and training on stewardship for farmers and veterinarians (25, 26). In the latest UK-VARSS report published in 2020, the data showed that antibiotic sales in food-producing animals halved since 2014 and over the same period, the use of Highest-Priority Critically Important Antibiotics (HP-CIAs) has reduced by 72% (27).

### Drivers and Barriers to Cross-Sectoral Collaboration on AMU/AMR Surveillance

Respondents identified two main drivers for cross-sectoral collaboration on AMU/AMR surveillance. Firstly, there was a recognition of the interconnectivity between humans, animals and the environment on AMR and the need for collaborative efforts to tackle it: “*The different surveillance activities,…some focus on humans, some focus on animals, and by integrating those data, that's really important, that's where you got the power from because AMR is something that can cross species borders, bacterial species borders, across different environments. So, it's really important to consider it as a whole to understand where it's coming from”* (P18). Secondly, the fact that this collaboration was a recommendation of the NAP meant that the different institutions had to implement it. A previous study that was conducted to evaluate of the implementation of the UK AMR strategy 2013–2018 found that the strategy has greatly improved communication and collaboration between human and animal health sectors (28). Interviewees highlighted also the synergies between human and animal sectors when tackling AMR: “*There's obviously solutions on the human side, and obviously solutions on the animal side and they're so intrinsically linked, so avoiding infection in the first place. Development of diagnostic tools, development of new medicines, you know, the language is very similar, the problems are similar, the solutions can be similar, the behaviour change aspect is very similar on both sides as well”* (P15).

In term of barriers to cross-sectoral collaboration, some interviewees considered that there were no obstacles because there is a common goal to be achieved. Others identified barriers with the main ones being resources and the differences in methods; previous studies reported the same obstacles to integrated surveillance (20, 21, 29, 30). The following quotes illustrate a participant' views regarding the availability of resources: “*So the issue with speaking of companion animals specifically, one of the major issues and barriers to collaboration is quite bluntly funding, or the lack there of”* (P17). In addition, the lack of resources specifically for cross-sectoral collaboration was highlighted: “*We are forced to prioritise more and more; the budget is being cut and our top priorities are not One Health. …not as a priority; it is an objective, yes, but when you drill down … that makes it then difficult if you haven't got dedicated resources” (P10)*. Competing priorities within organisations was also mentioned as a factor that could impact the involvement of some stakeholders in integrated surveillance.

Regarding the variations in the methods used, this was considered as a technical barrier because it hinders the comparison of data: “*…there are still huge limitations and how and whether we can overcome them, like standardisation of panels for example”* (P10). This variation in methodology was considered to be due to the way the different sectors have evolved.

### Need for More Cross-Sectoral Collaboration on AMU/AMR Surveillance

Some participants regarded the current level of cross-sectoral collaboration as adequate, whereas others mentioned a need for improvement. This was, perhaps not surprisingly, particularly prominent amongst interviewees who worked in the environment sector and small animals' sub-sector who did not have ongoing AMR surveillance programmes and therefore had less opportunities for collaboration. This might change in the future especially with the increasing recognition of the importance of having more data from these sectors. The main areas identified by respondents as requiring improvement include the following: (a) Methods; (b) Resources and capacity; (c) Improved coordination of integrated surveillance activities; (d) Surveillance data from the environment; (e) Surveillance data from companion animals; and (f) Communication and sharing of information.

#### Methods

Respondents mentioned that there is a need for improvement in the way the data are collected and analysed to allow comparison between the different sectors. Some interviewees called for a standardisation of AB panels between humans and animals to be able to compare the data; others considered that standardisation could have a negative impact on the current system: “…*if you standardise, you're forcing change on a lot of people who don't want change… So you're forcing change on a system that has evolved for a reason in a certain direction. It doesn't necessarily mean that system's going to get better*” (P16). The difficulty to achieve harmonisation between the different methodologies was also raised by some respondents: “…*how do you achieve harmonisation? Because people are very wedded to their own systems so how do you get them to change, you know? Especially because they've been collecting data for so long, they want to do their backward comparison and so, they don't want to move from that position to do a sideways comparison*” (P5). One of the possibilities proposed was to start a new system as it was the case for the EU harmonised monitoring programme, but then the problem that had emerged was the fact that it was not possible to compare with the other system in the same country. The need for more harmonisation and integration between sectors has been acknowledged in the UK NAP 2019–2024 (9) and respondents explained that work was ongoing to address the commitments from the strategy.

#### Resources and Capacity

There was an overall agreement that activities are driven by funding and several respondents commented on the need to have funding specifically for cross-sectoral collaboration: “*I also think funding opportunities that are specifically for working across sectors would also promote further working. You're providing additional resource to facilitate those cross workings, it would be really helpful*” (P18). Funding was also seen to enable some integrated activities to be conducted more frequently. As an example, when asked if there was a plan to produce the OH report more frequently, P10 said: “*I don't think so, it's very, very resource intensive. So, the original plan was to have it biannually, that has been obviously been delayed*” (P10). The importance of having funding dedicated specifically to cross-sectoral collaborative activities has been described in previous studies as key for OH initiatives to achieve their potentials (19, 29).

#### Improved Coordination of Integrated Surveillance Activities

Participants highlighted the need for better coordination of integrated surveillance for AMU/AMR with some proposing the establishment of a coordinating committee or group. When asked about who should oversee this coordination, respondents mentioned the need for a group which should have three main characteristics. Firstly, it was perceived that because it is a shared responsibility, this group would need to have representatives from all sectors. It was felt that if one sector takes the lead they would push for their own priorities. Secondly, this group needs to have the power to act: “*Ultimately, it has to be somebody in power, so I would say probably central government, and then responsibility divested down… The higher up you go and the higher up your responsibility, the more other players you'll get involved*” (P16). Finally, this group needs to define clear set of measurable outcomes: “*I think that would be my main recommendation to give it a proper mandate to have a committee, with clearly set targets because even in the national strategy it is rather vague; I think we do need measurable objectives*” (P10). Some participants proposed the use of existing group such as DARC to coordinate this collaboration instead of creating parallel groups: “*Well I, if you really ask me then I would mandate a group like DARC to do that, rather than creating another body,…it doesn't feel right to create a parallel committee*” (P10).

#### Surveillance Data From the Environment

It was widely acknowledged that there was a lack of surveillance data on AMR from the environment: “*…it's an area that's a big gap that we need to look at, the environment”* (P11). Research on environmental AMR was seen to have been mainly done by universities, which was perceived as important but would not replace the implementation of a statutory surveillance programme: “*Environmental AMR so far has been really an academic or a university topic, so funded by the research councils, and there's still plenty of things that need researching at those organisations or those institutions, but we also need to move on and do things for real, and universities cannot replace a government surveillance or those sort of things*” (P9). It was also considered that the environment sector could learn from the experience gained in other sectors since they had ongoing surveillance programmes for AMR in place for a long time. In addition, the need for funding to conduct the work was also mentioned. Interviewees highlighted that this is an area where all sectors need to collaborate because antibiotic usage from humans and animals ends in the environment: “*The environment goes across all sectors and that's one of the big areas where we should all be collaborating on. Because whether it's human health, companion animal or farm, the environment is where it all ends up*” (P14).

#### Surveillance Data From Companion Animals

Respondents highlighted the importance of continuous monitoring of AMU and AMR from companion animals to better understand the risk from this animal category. Currently, antibiotic usage data from companion animals are not systematically collected but the VMD funded studies to investigate it and the results were included in the UK-VARSS reports (31–33). These studies looked into AB prescriptions using the data that were extracted from practise management systems by VetCompass and the Small Animal Veterinary Surveillance Network (SAVSNET) system that are managed by the Royal Veterinary College and the University of Liverpool, respectively. Antibiotic sales data for dogs and cats are published in the UK-VARSS reports. Building on the success of the TTF, RUMA announced the formation of RUMA Companion Animal and Equine Group, which will draw from the learnings from the TTF to develop sector specific goals to drive improvements.^6^

Little collaboration was reported between human sector and companion animals' sub-sector, which was explained by the lack of understanding of the value of examining AMU and AMR data in companion animals: “*Very little collaboration with human side, currently yeah. Not because we're not interested in doing it, it's just because it's not happening, and there isn't a lot of interest coming back. I guess the human side would have to start to understand that there is a value in exploring usage in companion animals, and that currently isn't really happening*” (P16). Reflecting on the fact that there is little interest in data from companion animals compared to food producing animals, P17 said: “*…there's quite a heavy legislation surrounding food safety, therefore resistance goes higher up the priority tree, because they've legislative duty or responsibility where to ensure the food is safe. Whereas companion animals, there isn't really any legislative work aside from the relatively vague animal welfare laws*.”

This lack of collaboration was also seen to be due partly to the fact that there is little awareness of the AMR risk from companion animals and a first step would be to raise awareness on the issue: “*A lot of owners I suspect aren't even aware that there are issues with antimicrobial resistance in companion animals. So there's a huge pile of work that could be done just on raising awareness, but that would have to come from the human side*” (P16). Respondents mentioned also the need to better understand the different pathways of transmission of AMR between companion animals and humans that are completely different to those between humans and livestock: “*The whole storey with humans and companion animals is much more complex, because a lot of animals will lick owner's faces, …owners will hold them, they will sleep in the bed with them. Their lives are entwined around animals. Obviously then resistance can be passed on by multiple different routes”* (P16).

#### Communication and Sharing of Information

Respondents highlighted the importance of improving collaboration by better communication and sharing of information: “*I think there's always scope to improve collaboration, better communication, better sharing of information, more timely sharing of information and having reactive systems that you don't have to wait for the next committee, you've got a mechanism to react to sudden findings of concern*” (P6). An example that was mentioned by one informant was the importance of communication with veterinarians about some resistance mechanisms circulating in companion animals that are of relevance to humans: “*I suppose just also sort of better communication as well so that people are aware that these resistance mechanisms may also be found in veterinary samples*” (P12). A wider understanding of how the different sectors work was also seen as an important outcome for an improved communication.

It was also highlighted that work was ongoing to explore options to use AMR data from animals generated by private laboratories. Currently, these data are not accessible and only some resistance data from small animal private laboratories are collected through the Small Animal Veterinary Surveillance Network (SAVSNET)^7^. The following quote illustrate this: “*The private labs, for example, we are looking into developing that and work more closely with the private sector and explore options to work more with them, because obviously there are private labs that do testing for resistance. And we know that their data is available but currently we don't have clear access to that data, so we are looking into it*” (P1). Increasing the availability of AMR data from clinical samples in animals through collaboration with private laboratories would have an impact in increasing the sensitivity of the surveillance system to detect trends in AMR circulating at the human-animal interface (6).

## Discussion

In this study, we aimed to evaluate qualitatively the performance and value of integrated surveillance system for AMU/AMR in England by applying the ISSE framework. The results showed that there are links between integrated surveillance information, decision making and intervention; especially in the case of the Res-Alert programme. However, there were only few OH examples where the potential of collaboration was fully exploited. Many benefits described were related to the generation of information and increase in knowledge and understanding without explicit use of the information for policy or intervention development. These intangible benefits have a value but being able to link surveillance information and mitigation measures is very important as it would help to enhance the value of integrated surveillance efforts. This is particularly relevant considering budget constraints and the need to justify resource allocation to activities. Integration and collaboration are resource consuming and full integration in a system might not be necessary to achieve the wanted outputs. Therefore, it is important to identify the level of collaboration that will achieve the optimal performance and cost-effectiveness (30, 34). In the following paragraphs, the results presented in the previous section are discussed using the levels of the ISSE framework.

### OH Integration in the Surveillance System

This study showed that there were various integrated surveillance activities for AMU and AMR with various modalities and degree of integration and the types of outputs and outcomes depended on the type of activity. The main integrated activities reported that had a formal structure were the AMR contingency plan, the UK OH reports, and the DARC group. Some of the collaborative activities that were mentioned were informal based on the network of contacts. Although this informal networking has a (perceived) value, their long-term benefit is uncertain, because collaborations may cease if the people involved move on and there are no mechanisms to promote institutional or network memory. Another special type of collaboration mentioned was the TTF, an example of public-private partnership. Although the TTF was formed mainly by stakeholders from the animal health sector, the FSA attended as an observer and an independent scientific group with experts from different sectors provided advice to the group.

With regards to governance, the surveillance system for AMU/AMR is embedded in the UK NAP for AMR and the recommendations from the NAP are used as levers to implement collaborative surveillance activities. The NAP is coordinated by the HLSG but there is no committee for the coordination of integrated surveillance activities. Respondents commented on the need for better coordination of integrated surveillance activities and the formation of a cross-sectoral group to oversee this. It is important to have adequate governance modalities for steering and coordinating integrated surveillance to define the collaborative strategy that would achieve the objective of this surveillance and to provide necessary guidance and resources for its implementation (30). In addition, this committee can also be a platform for discussions on how to overcome the barriers to cross-sectoral collaboration such as the difference in methods used between sectors. Recently, a OH integrated surveillance (OHIS) sub-group of DARC was formed with the aim to strategise the integration of AMR surveillance across the different sectors and the four nations^8^.

While there is no structured mechanism for data and information sharing between sectors, a lot of information that is produced is available in the public domain (e.g., the UK-VARSS and the ESPAUR reports; and the online data portal “Fingertips”). Having these data publicly available increases transparency and presents an important source of information for stakeholders, including policymakers, the livestock industry, and other interested actors. There is work ongoing to explore options for improved sharing of AMR surveillance data between sectors in the UK such as the development of a shared database^9^ This would enable the collation and analysis of data to be developed into an ongoing activity, which would improve the timeliness of data availability and enables rapid identification of emerging trends; therefore improving the effectiveness of integrated surveillance (35). Furthermore, this shared database can serve as a resource for risk assessment and generation of evidence for policies to protect animal and human health. An example of this is the DANMAP database which collects data from several sources including food and hospital laboratories, slaughter plants, veterinary practises and general practises for people. These data are collected from various microbiological laboratories that use different data formats and therefore these data are imported, merged and cleaned before conducting the data analysis (35, 36). At global level, the UK contributes to international surveillance, which was considered by interviewees as beneficial and adding value to the data. At local level, we found that the first regional OH group on AMR in the UK was successful at the beginning with various outputs produced, which helped to facilitate discussions and enabled the sharing of knowledge and ideas (22). However, there was limited success for the group to maintain the work, with the main barrier reported by interviewees being the lack of funding for these regional groups.

There is no funding available specifically to integrated surveillance on AMU/AMR and participants highlighted that this constitutes a constraint to cross-sectoral collaboration. In addition to the resources needed for sectoral surveillance, specific resources must be allocated for activities involving several sectors both at the governance and at operational levels (30). Integration can take place at different steps of the surveillance and to various extent and full integration might not be necessary to achieve the desired outputs. Therefore, it is important to identify the degree of collaboration that will achieve the OH surveillance objective in a cost-effective manner (30). This can be achieved for example by conducting economic evaluations of different levels of surveillance integration (linked to interventions) and assess the benefits they generate. However, it is important to consider not only tangible benefits but also intangible ones. Intangible benefits such as knowledge creation, social and intellectual capital, peace of mind, political and technical reassurance are difficult to capture through quantitative metrics in the short term but can lead to large benefits in the long term.

### Production of OH Information and Expertise

Various integrated surveillance activities were identified and within these there were various OH teams working to produce outputs. Some members were involved in multiple integrated activities but others were involved only in one. For example, the OH team producing the UK OH reports was formed by representatives from the different sectors that had good working relationship and worked well as a team based on the comments from interviewees. Similarly, the OH team of the DARC group was considered as a good team with the right people in it. For the AMR contingency plan, members who are managing the alert system are the same but other members change based on the hazard identified. Regarding the OH network, surveillance data produced are publically available so can reach a wide range of stakeholders. In term of the quality of OH information produced, the current surveillance system includes data from humans, animals and food, but does not include data from the environment. However, work is ongoing to include data from this sector (9). Respondents highlighted also the power of this collective data in providing a stronger message and sounder evidence. The importance of information generated by OH surveillance activities to better inform discussions in terms of risk assessment and management in the case of zoonosis disease has been described previously (34).

There is also ongoing work to strengthen sectoral surveillance components by collecting AB usage data from species that are not covered and by increasing coverage in species with low coverage; and by exploring options to include AMR data in animals from private laboratories. Strengthening sectoral surveillance components will have an impact on improving the outputs and outcomes of integrated surveillance (2, 4).

### Generation of Actionable Knowledge and Influence on Decision Making

The outcomes of integrated surveillance activities varied depending on the type of the activity. For example the AMR contingency plan was considered by respondents as an action oriented activity allowing to detect emerging resistances. It was clear from participants' views and examples provided that the information and evidence generated by this integrated activity was shared with relevant stakeholders and was used to make relevant decisions at policy level for interventions to be implemented. This was a direct example of how OH information produced impacted on decision making. For the UK OH reports, however, the main outcomes were about improving integrated analysis and harmonising the data across sectors, which in turn help to better understand the epidemiology of AMR and detect AMR trends at the human-animal interface. These reports are publically available and are valuable sources of information that can be used by stakeholders for a better understanding of cross-sectoral issues on AMR. However, it was not possible to evaluate the impacts of this OH information produced.

Regarding the DARC group, the main outcomes were related to communication and exchange of information and expertise, which leads to an increase in awareness and understanding of the AMR issue and its wider impacts. In addition, cross-sectoral communication was considered key in understanding other sectors' priorities and challenges and enabling discussions to reach agreeable decisions. Another positive outcome was the establishment of good working relationships across sectors and relationships that could be used for further collaborative work. The improvement of knowledge and understanding and generation of social and intellectual capital have been identified as benefits of OH approaches in reviewed literature (37). These intangible benefits should be considered in evaluation in addition to monetary outcomes although its quantification is not always possible (2, 34).

### Contribution to Desirable Outcomes

There has been a decrease in AB sales in food-producing animals in the UK in the past years, which made the UK one of the lowest users of antibiotics across Europe (27, 38). This decrease was mainly due to the work of the RUMA TTF, farmers and the different stewardship programmes developed by the different livestock sectors (25, 27). Following this, a decrease in the levels of resistance in *E. coli* isolates from broilers examined at slaughter was observed for most antibiotics tested (16). Also, resistance in in *E. coli* from healthy pigs at slaughter was reported at lower levels compared to five years ago (27). These data show real progress and possible impact of the reduction of AMU in animals but long-term monitoring is needed to be able to fully assess these links, especially that it can be challenging to differentiate between the benefits of cross-sectoral collaboration and the wider benefits of surveillance.

In terms of improvement, the main areas mentioned were the development of more harmonised methods for data collection and analysis across sectors, better coordination of integrated surveillance, having resources dedicated to cross-sectoral collaboration, and collection of surveillance data from the environment and from companion animals. The use of comparable methods is necessary to allow comparison of results not only within the country but also within region and at global level. The guidance developed by WHO states that a programme of integrated surveillance of AMR in foodborne bacteria needs to include coordinated sampling and testing of antimicrobial susceptibility of bacteria from food-producing animals, food and humans using epidemiological (including sampling) and microbiological methods that enable comparisons of results (39). With regards to surveillance data from companion animals, there is a need for continuous monitoring of AMU and AMR from this category to better understand the risk that they pose. Although the quantity of antibiotic use in this category is low compared to other farm animals, there is a high risk due to their close interactions with humans. However, there is little awareness of this risk and participants highlighted the importance to increase awareness about this issue. On the environmental side, respondents highlighted the huge data gap and the need for greater integration. The knowledge and evidence gap of AMR in the environment has been increasingly recognised (40, 41) and one of the of the priorities in the new NAP is to better understand the role of the environment in the spread of AMR (9). The implementation of changes in such a complex system can be challenging especially that surveillance is expensive and appropriate resources need to be allocated to integrated activities. However, having a NAP which includes commitments to develop a more harmonised and integrated surveillance for AMU/AMR can help in directing the necessary resources and capacity to achieve the goals set.

An important dimension linked to integrated surveillance that was identified in this study is cross-sectoral collaborative research, with several examples of activities involving different sectors. Research was considered to underpin surveillance work and targeted research works fill the gap in knowledge and allow the development of new methods that could be used in surveillance. Hence, it is important to assess the capacity of the surveillance system to facilitate research work and this is another dimension that could be added to the ISSE framework. Another dimension that could be added to this framework relates to the collaboration at international level. The importance of collective data was highlighted by many participants and it is important to assess this explicitly when conducting the evaluation.

Several of the benefits described by participants were intangible such as enhanced knowledge and understanding, and social capital. Future studies should aim to capture these benefits explicitly. Moreover, the associations between outputs and outcomes were not always clear. Several integrated activities were described with some producing OH information which impacted on decision making, while others producing outcomes related mainly to the generation of information and increase in knowledge without links to how the information generated is used. With firm links between surveillance information and mitigation measures with clear attribution, this would allow estimating the value of integrated surveillance efforts with more accuracy. In addition, this would enable to better recognise the benefits of cross-sectoral collaboration, as it is currently challenging to differentiate between the wider benefits of surveillance and the benefits of collaboration.

The ISSE framework used is new and this study was one of its first applications. This framework was a useful foundation to structure the evaluation of different outcomes in the case of integrated surveillance for AMU/AMR. However, the framework does not provide guidance on how to conduct the evaluation, which data to collect and how to analyse them; this needs to be elaborated by the evaluator(s). It is comprehensive and encourages consideration of a broad range of elements and change pathway. When considering all five evaluation levels, as done in this study, a lot of data and time are required to do the evaluation. Resources can potentially be saved if users focus only on one or two levels depending on their needs and context. This framework can also be used in combination with other tools such as NEOH and ECoSur.

Purposive sampling is widely used in qualitative research to select information-rich cases related to the phenomenon of interest (42). In this study, purposeful sampling was used to identify stakeholders who have knowledge, experience and involvement in the surveillance system for AMU/AMR in England covering human, animal, food and environmental sectors; which allowed to include diverse viewpoints. Although we covered key stakeholders involved, not all groups were included such as practitioners, representatives from relevant industries and Non-Governmental Organisations. Purposively selected samples are of benefit for qualitative research as it allows the collection of rich, in depth account from participants.

This evaluation allowed to identify effective and ineffective points in the surveillance system for AMU/AMR in England. The effective points include: (1) the presence of a National Action Plan based on a OH approach with commitments to strengthen integrated surveillance for AMU/AMR; (2) the presence of a cross-sectoral alert system which allows a OH response in the case of identification of resistance bacteria or genes posing high risk; (3) the presence of cross-sectoral working groups; (4) publication of surveillance data in reports that are publically available; and (5) production of OH joint reports that are publically available. These activities demonstrate a commitment to One Health in AMU and AMR surveillance and lead to positive outcomes such as the provision of enhanced information for decision making, improved communication, exchange of information and expertise, and increase of awareness and understanding. Inefficiencies in the system identified were the absence of a steering and coordinating committee to oversee integrated surveillance activities; the lack of resources dedicated specifically to integrated surveillance activities for AMU/AMR; and the variations in the methods used between sectors. This evaluation showed also that the surveillance system in England is evolving with various initiatives recently implemented or under development. Examples of these activities include the formation of a OH integrated surveillance sub-group of DARC, collection of AB usage data from companion animals, collection of AMR data from the environment and from private laboratories, and the development of a database to share AMR data across sectors. These initiatives aim to enhance the availability of AMU and AMR data, and improve data sharing and collaboration. This indicates that the stakeholders are implementing changes and making investments in OH to address the needs identified. It is expected that with additional data becoming available, more evidence will be generated which will enhance the understanding of the epidemiology of AMR at the human-animal-environmental interface; and therefore inform the implementation of evidence based integrated surveillance activities on AMU/AMR. However, it is important to have regular evaluation to ensure that the surveillance programmes are operational and effective.

This study allowed an understanding of the capacity of the system to produce OH surveillance information and the links between this OH information produced and the various outputs and outcomes. Based on this evaluation, we propose the following indicators that can be used for the assessment of the performance of integrated surveillance system for AMU/AMR. These are: (i) the capacity of the system to produce OH information; (ii) the capacity of the system to use the OH information generated to enhance the knowledge and inform the implementation of interventions; and (iii) the capacity of the system to provide a OH response in the case of identification of resistance bacteria or genes posing a high risk to human or animal health. Future work on surveillance evaluation should consider ways of measuring these three indicators. The lack of knowledge on the most effective and efficient integrated surveillance strategies for AMU/AMR makes the development of benchmarks and best practises challenging. Research on how the different levels of integration influence the performances of integrated surveillance systems for AMU/AMR would allow to generate the evidence needed for recommending best practises. This study contributed to this knowledge and more will be learned once other integrated surveillance systems are evaluated and more evidence is generated.

## Data Availability Statement

The datasets generated and analysed during the current study are not publicly available due to the non-anonymised nature of the data. Requests to access the datasets should be directed to hbennani@rvc.ac.uk.

## Ethics Statement

The studies involving human participants were reviewed and approved by the Social Sciences Research Ethical Review Board (SSRERB) at the Royal Veterinary College, with the approval number URN SR2019-0204. The patients/participants provided their written informed consent to participate in this study.

## Author Contributions

HB conceptualised the study and data collection protocols with guidance from BH, LC, and KS. HB was responsible for data collection, analysis, and the drafting of the first version of the manuscript, with BH directly involved and providing inputs to all the work. All authors provided feedback and inputs and critically reviewed all manuscript drafts. All authors approved the final version of the manuscript.

## Funding

This work was conducted as part of a Ph.D. study funded by the Bloomsbury Colleges Ph.D. Studentships Programme.

## Conflict of Interest

The authors declare that the research was conducted in the absence of any commercial or financial relationships that could be construed as a potential conflict of interest.

## Publisher's Note

All claims expressed in this article are solely those of the authors and do not necessarily represent those of their affiliated organizations, or those of the publisher, the editors and the reviewers. Any product that may be evaluated in this article, or claim that may be made by its manufacturer, is not guaranteed or endorsed by the publisher.

## Footnotes

^1^https://guidance.fp7-risksur.eu/.

^2^https://fingertips.phe.org.uk/profile/amr-local-indicators.

^3^https://eur-lex.europa.eu/legal-content/EN/TXT/PDF/?uri=CELEX:32020D1729&from=EN.

^4^https://assets.publishing.service.gov.uk/government/uploads/system/uploads/attachment_data/file/479015/LA-MRSA.PDF.

^5^https://assets.publishing.service.gov.uk/government/uploads/system/uploads/attachment_data/file/518845/LA-MRSA_abattoir_leaflet.pdf.

^6^https://www.ruma.org.uk/new-medicine-initiative-for-pets-and-horses-draws-on-farm-livestock-learnings/.

^7^https://www.liverpool.ac.uk/savsnet/.

^8^https://assets.publishing.service.gov.uk/government/uploads/system/uploads/attachment_data/file/994935/PCDOCS-_2080209-v1-DARC_Summary_Minutes__4_3_21_.pdf.

^9^https://www.food.gov.uk/news-alerts/news/ps192-million-for-cross-government-surveillance-project-to-protect-public-health.
