# Supplementary material for: Evaluating Integrated Surveillance for Antimicrobial Use and Resistance in England: A Qualitative Study
Source: Front Vet Sci. 2021 Nov 2;8:743857. doi: 10.3389/fvets.2021.743857 (PMC8596565; doi:10.3389/fvets.2021.743857)
Supplement: Supplementary file 1 [file Data_Sheet_1.DOCX]

Supplementary Material

# Supplementary material A: Interview guide

Introduction and consent

About your involvement on AMU/AMR surveillance

1. Integrated surveillance activities for AMU/AMR in England

- Can you tell me what surveillance systems or surveillance component for AMR and AMU you are in charge of?
- Can you describe any work that you do with other sectors on AMR and AMU surveillance?
- Which sectors and disciplines? (Humans, animals, food and the environment; different disciplines)
- What are the driving reasons for working with other sectors or disciplines on AMR/AMU surveillance? What do you hope to gain from the collaboration? Are there barriers and levers?
- How is this collaboration organized? Is there a formal agreement about how to collaborate and which stakeholders to be involved?
- Is there a common One Health (OH) objective formulated for AMR and AMU surveillance? If yes, what is it? If no, do you think it is important to have one?
- Are there funds available specifically to cross-sectoral collaboration on AMR and AMU surveillance?
- If yes, how much and how they are used? Is it enough? What would this needs to be ideally?
- If no, do you think it should be?
- Have you ever evaluated the surveillance for AMU/AMR from an integrated perspective?
- If there is no collaboration, ask why? Should there be? Are you aware of any changes planned for the future?
- Is there any collaboration at the level of planning/designing the surveillance systems for AMU/AMR?
- Is there any collaboration with other sectors at the level of data collection, analysis and interpretation? At the level of data management and storage?
- Are there any surveillance activities in humans that are triggered by signals from data collected in animal surveillance?
- If yes, what are they?
- If no, what these should be? Do you consider these to be useful?
- Is there a formal structure/team/committee in charge of comparing AMU and AMR data between sectors?
- Is reporting of data analysis standardised to allow end users to compare between sectors? In what way?
- Are mechanisms in place to ensure data quality? What are they? Are they the same across all actors and institutions?
- Are there any mechanisms for steering and coordination of cross-sectoral collaboration?
- If yes, how does it work?
- If no, do you think it should be?

1. **Links between integrated surveillance activities and outputs**

*Builds on examples mentioned in the previous section about OH integrated activities for AMR and investigate if these integrated activities produces outputs in term of OH information, OH team, OH network or any other outputs and how effective they are.*

- You mentioned collaborative activities such as…, Can you tell me about the outputs produced by these OH integrated activities?

***Prompts:***

- How well does the system produces OH information? Are data included from the different sectors? Are appropriate methods used for integrated analyses, interpretation and dissemination?
- How important and effective is OH network? Are multiple and relevant organisations and expertise represented? How is communication between within the network?
- How effective is the OH surveillance team? Are multiple and relevant expertise represented? How is assessment of the collaborative work)
- Any other outputs?

1. Links between outputs and outcomes

*Builds upon examples given in the previous section and ask the participants to reflect on the effect of these OH outputs mentioned on the surveillance system immediate outcomes*

- How do you see the benefits of such integrated approaches to surveillance across sectors?
- Is there an impact of this collaboration on learning and knowledge acquisition? Can you give examples?

***Prompts:***

- Does OH collaboration contribute to enhance system understanding of AMR epidemiology at the animal-human-environment interface (capacity of the system to generate a better understanding)?
- Does OH collaboration contribute to detect trends and correlations between humans and animals when they exist? If yes, can you give examples?
- Does OH collaboration contribute to increase awareness in different stakeholders? (use of appropriate dissemination methods and strategies)
- Any other effects?

1. Impact of surveillance information on decision making

*Asks respondents how information produced by OH surveillance is used by different stakeholders and identify the decisions that have been taken based on this information and that can be attributed to the integration of the system*

- How does this cross-sectoral collaboration impacts on decision making? Can you give examples from your experience? (how information produced by integrated surveillance activities has been used by you or other stakeholders you work with?)

***Prompts:***

- Policy change regarding AMU/AMR: legislation on AMU in agriculture and humans, enhanced surveillance, awareness programs, etc..)
- Behaviour change in veterinarians, producers, distributors, consumers and the general public (AMU, consumption of animal products, societal values, etc..)
- Interventions

1. Impacts of the decisions attributable to OH integration

*Builds upon examples given in the previous section on decisions attributable to OH integration in the surveillance system and explore their impacts on the reduction of AMU and AMR in humans, animals and the environment.*

- You have mentioned …….,in your opinion what are the impacts of these decisions (interventions, no intervention, policy change, behavior change,…) on the reduction of AMU and AMR in humans and animals?
- What are the impacts on the health of humans, animals and the environment?
- To what extent we are able to link changes or interventions in the veterinary side and how that would impact on public health? Do we have the data needed to make these associations?

1. Sharing infrastructure

- Are there AMU or AMR data and/or information sharing mechanisms in place across different sectors?
- If yes, what do they look like (e.g. newsletters, workshops, reports available to all, results getting published, online information sharing platform….)? Formal/informal? What exactly is shared and at what level? Are there formal agreement in place? What are they?
- If yes, have you experienced challenges or barriers to data and/or information sharing across sectors? What are they?
- If yes, is there any added value of doing so? Can you describe it? Any examples of how additional information resulting from data and/or information sharing has been used to change/continue/stop an initiative.
- If not, why? Should there be?
- How accessible are the data?
- Are mechanisms/procedures in place to ensure safe and appropriate data accessibility to facilitate sharing? (e.g. is extraction of data feasible without access to experts, or are experts readily available for extraction of data, is the process of data extraction bureaucratic/overly time consuming?)
- Have resources been allocated specifically to data and information sharing?

1. Need for improvement

- Do you see any need for improvement of the cross-sectoral collaboration for AMU/AMR surveillance?
- If yes, in what way?

Concluding discussion

- Is there anything else you would like to add to what we have talked about today?
- Is there anyone that it would be especially important for me to talk to at this point?
- If at a later stage any questions emerge, can I contact you to follow up on some points?
- Thanks and close

# Supplementary material B: Roles of interviewees

*Table 1: Information about the roles of interviewees*

| **Interviewee reference** | **Role of interviewee** |
| --- | --- |
| P1 | Senior Officer |
| P2 | Senior Scientist |
| P3 | Professor |
| P4 | Senior Scientist |
| P5 | Senior Scientist |
| P6 | Senior Officer |
| P7 | Senior Officer |
| P8 | Senior Officer |
| P9 | Senior Scientist |
| P10 | Senior Scientist |
| P11 | Senior Scientist |
| P12 | Senior Scientist |
| P13 | Pharmacist |
| P14 | Senior Staff |
| P15 | Academic/Senior Officer |
| P16 | Senior Lecturer |
| P17 | Postdoctoral Scientist |
| P18 | Senior Researcher |

# Supplementary material C: Integrated surveillance activities

Key integrated surveillance activities reported by interviewees include the followings:

1) The Res-Alert contingency plan, which was considered as one of the main example of collaboration across sectors. This refers to the response upon identification of a resistant bacterial isolate from an animal considered to pose a potential risk to human and/or animal health. This is a UK wide plan initiated in 2015 and coordinated by the Veterinary Medicines Directorate (VMD) in collaboration with government agencies covering human, animal, food and the environment; and the Devolved Administrations. This contingency plan has four pillars, which work in an iterative process that are Risk Alert, Risk Assessment, Risk management and Risk communication. Depending on the hazard identified, relevant advisory committees are notified by the relevant agencies and the actions that are taken depend on the outcomes of the discussions. Food Standards Agency alerts the Advisory Committee on the Microbiological Safety of Food (ACMSF) sub-group on AMR if the resistance is detected in a pathogen suspected to be foodborne; VMD to alert the DEFRA antimicrobial resistance coordination (DARC) group; DHSC to alert the Advisory Committee on Antimicrobial Prescribing, Resistance and Healthcare Associated Infection (APRHAI); PHE to alert the Human Animal Infections and Risk Surveillance (HAIRS); and VMD to alert the UK Zoonoses, Animal Diseases and Infections (UKZADI);

2) The UK One Health reports on antibiotic use and resistance were produced by PHE and the VMD in 2015 and 2019. In the second OH report, in addition to the data included in the first one, data on AMR in isolates from retail meat was included and also the UK results for the EU Harmonised key outcome indicators for humans and food producing animals (FPAs) were presented. These indicators were developed by the European Centre for Disease Prevention and Control (ECDC), European Food Safety Authority (EFSA) and the European Medicines Agencies (EMA) for monitoring of antibiotic consumption and resistance in EU member states;

3) Advisory committees in one sector with representatives from other sectors such as the DARC group; The Advisory Committee on Antimicrobial Prescribing, Resistance and Healthcare Associated Infection (APRHAI); and the Advisory Committee on Microbiological Safety of Food (ACMSF) subgroup on AMR.

4) The Responsible Use of Medicines in Agriculture Alliance (RUMA) Target Task Force (TTF), is a group that was formed in 2016 to develop sector specific targets for the key UK livestock sectors. The group comprises a specialist veterinarian and leading farmer or sector representative for each key UK livestock sectors (beef, dairy, calves, sheep, pigs, salmon, trout, gamebirds, laying hens and poultry meat); with observers from the VMD and Food Standards Agency (FSA) . This is a voluntary initiative which was set up to develop sector specific targets (2017-2020) focusing on the reduction of antibiotic use in animals through measuring accurate usage, promoting prudent antibiotic use principles and optimising disease management. Building on the success of this first task force, the TTF2 was formed to set targets from 2021 to 2024. In addition, a sister group to RUMA was set up for companion animals and horses to promote responsible use in these species. In addition, an Independent Scientific Group with scientific experts from different sectors was formed to provide advice to the group.

Examples of other integrated activities reported by participants include: a) Collaboration between the two main government (PHE and APHA) laboratories to exchange information about cases and compare isolates; b) Joint in conferences; and c) Collaboration between APHA and the British Society for Antimicrobial Chemotherapy (BSAC) by meeting medical colleagues and discussing testing issues.
